# Supplementary material for: A remarkable genetic shift in a transmitted/founder virus broadens antibody responses against HIV-1
Source: eLife. 2024 Apr 15;13:RP92379. doi: 10.7554/eLife.92379 (PMC11018346; doi:10.7554/eLife.92379)
Supplement: Figure 3—source data 1. [file elife-92379-fig3-data1.zip › Figure 3 ΓÇô source data 1 /Uncropped western blots for Figure 3B.pptx]

## Slide 1
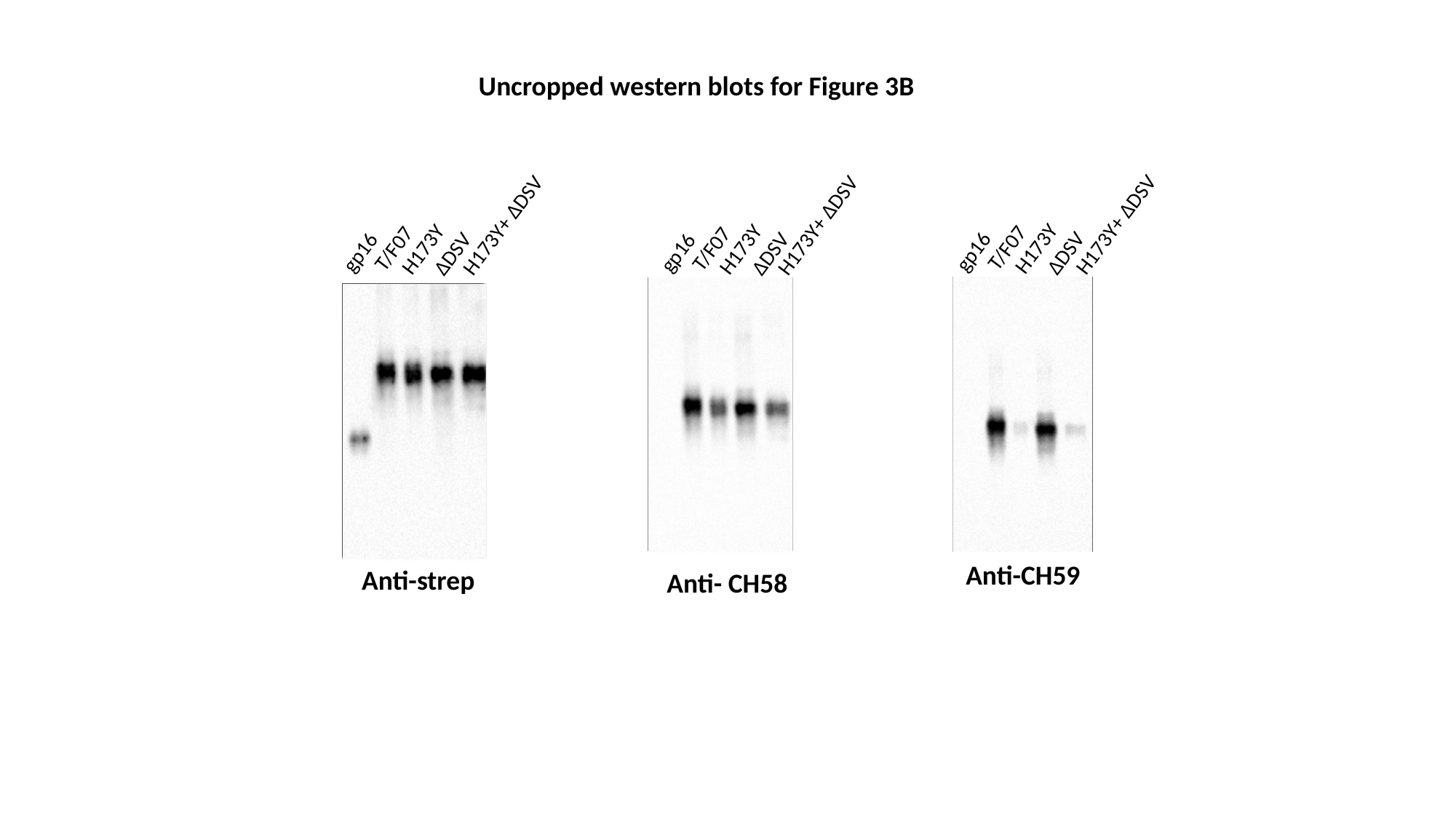

Uncropped western blots for Figure 3B
H173Y+ ∆DSV
H173Y+ ∆DSV
H173Y+ ∆DSV
gp16
gp16
gp16
T/F07
T/F07
T/F07
H173Y
H173Y
H173Y
∆DSV
∆DSV
∆DSV
Anti-CH59
Anti-strep
Anti- CH58
