# Supplementary figures and images for: A remarkable genetic shift in a transmitted/founder virus broadens antibody responses against HIV-1
Source: eLife. 2024 Apr 15;13:RP92379. doi: 10.7554/eLife.92379 (PMC11018346; doi:10.7554/eLife.92379)

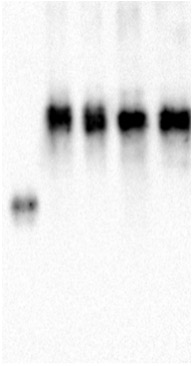

Supplement: Figure 3—source data 1. [file elife-92379-fig3-data1.zip › Figure 3 ΓÇô source data 1 /Anti strep tag blot.jpg]

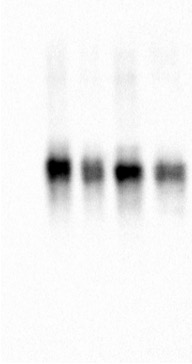

Supplement: Figure 3—source data 1. [file elife-92379-fig3-data1.zip › Figure 3 ΓÇô source data 1 /Anti CH58 Blot.jpg]

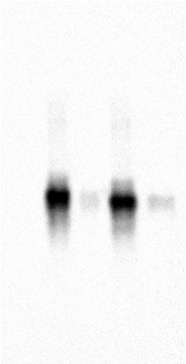

Supplement: Figure 3—source data 1. [file elife-92379-fig3-data1.zip › Figure 3 ΓÇô source data 1 /Anti CH59 Blot.jpg]
